# The Effect of Mobile Payment on Payment Waiting Time for Outpatients With Medical Insurance: Historically Controlled Study

**DOI:** 10.2196/43167

**Published:** 2023-01-26

**Authors:** Wanhua Xie, Xiaojun Cao

**Affiliations:** 1 Outpatient Department Guangzhou Women and Children's Medical Center Guangzhou Medical University Guangzhou China; 2 Guangdong Provincial Clinical Research Center for Child Health Guangzhou China; 3 Department of Science, Education and Data Management Guangzhou Women and Children's Medical Center Guangzhou Medical University Guangzhou China

**Keywords:** smartphone, mobile payment for health insurance, waiting time, outpatient service, patient satisfaction, medical insurance, mobile payment, mobile app

## Abstract

**Background:**

Waiting for a long time to make payments in outpatient wards and long queues of insured patients at the checkout window are common in many hospitals across China. To alleviate the problem of long queues for payment, many hospitals in China have established various mobile apps that those without health insurance can use. However, medically insured outpatients are still required to pay manually at the checkout window. Therefore, it is urgent to use information technology to innovate and optimize the outpatient service process, implement mobile payment for medically insured outpatients, and shorten the waiting time for outpatients, especially in the context of the COVID-19 epidemic. Furthermore, smartphone-based mobile payment for outpatients with health insurance could be superior to on-site cashier billing.

**Objective:**

This study aimed to investigate the impact of smartphone-based mobile payment in relation to different aspects, such as waiting time, satisfaction with patients’ waiting time, payment experience, the proportion of those dissatisfied with payment, total outpatient satisfaction, and outpatient volume, and compare mobile payment with on-site payment.

**Methods:**

This was a historically controlled study. This study analyzed the outpatients’ waiting time to make a medical insurance payment, their satisfaction with the waiting time and payment experience, the proportion of those dissatisfied with payment, and the outpatient volume of patients at Guangzhou Women and Children’s Medical Center 1 year before and after the implementation of mobile payment for medical insurance in January 2021. An independent sample 2-tailed *t* test was used to compare waiting time, satisfaction with waiting time, and overall satisfaction. Paired sample 2-tailed *t* test was used to compare monthly outpatient visits. The chi-square test was used to compare the percentages of patients dissatisfied with payment.

**Results:**

After the implementation of mobile payment for medical insurance outpatients, the patients’ payment waiting time was significantly shortened (mean 45.28, SD 10.35 min vs mean 1.02, SD 0.25 min; *t*_9014_=53.396; *P*<.001), and satisfaction with waiting time and payment experience were significantly improved (mean 82.08, SD 3.17 vs mean 90.36, SD 3.45; *t*_9014_=–118.65; *P*<.001). Dissatisfaction with payment significantly decreased (10.27%, SD 2.18% vs 1.19% vs SD 0.30%; *P*<.001). The total satisfaction of outpatients significantly improved (mean 86.91, SD 3.23 vs mean 89.98, SD 3.31; *t*_9014_=–44.57; *P*<.001), and the outpatient volume increased (248,105.58, SD 89,280.76 vs 303,194.75, SD 53,773.12; *t*_11_=2.414; *P*=.03). Furthermore, payment efficiency improved, and the number of the on-site cashiers substantially decreased.

**Conclusions:**

Mobile payment for health insurance significantly shortened patients’ payment waiting time; improved patient satisfaction on waiting time and payment experience and overall satisfaction; reduced the proportion of patients who were dissatisfied with payment and the cashier at the hospital; and increased monthly outpatient volume. This approach was effective and thus worthy of promoting.

## Introduction

### Background

The excessive waiting time in outpatient clinics is regarded as a major problem in health care management worldwide [1,2]. Hospital waiting time and payment methods influence outpatient flow and satisfaction [3]. Previous studies have reported that long waiting time for a medical consultation has become an important factor affecting outpatients’ satisfaction [4,5]. It is well-known that social disparities exist in the realm of medical care. The cost structure of a mandatory insurance scheme in a low-income country differs substantially from high-income settings. Replication studies for other countries are warranted [6]. In many tertiary hospitals with many outpatients in China, the phenomenon of long queues in front of the checkout window are very common. Under the traditional model, patients are required to pay for medical consultations at the cashier window or through self-service machines [7]. Generally, there are 3 to 4 kinds of outpatient expenses, including registration fee, inspection fee, examination fee, and medicine collection fee. Patients are required to queue at the corresponding window to make payments, leading to a long lineup in front of the cashier window, especially in hospitals with many outpatients. In the meantime, it takes a lot of time for patients, and their families waste a lot of time constantly going back and forth between the cashier window and different departments. In low-income countries, not all outpatients have medical insurance, and even those with medical insurance have to pay a certain percentage of the cost, which is paid by the patient. In outpatient clinics in China, some patients pay their own expenses, some have medical insurance, some receive free medical treatment, pregnant women may have maternity insurance, etc. The reimbursement ratio of medical insurance outpatients ranges from 40% to 60%, and the reimbursement ratio varies with different drugs. Only after uploading the patient data to the Guangzhou Medical Insurance Bureau information system would a patient learn how much they would be reimbursed and how much they would have to pay by themselves. The reimbursement limit for drug expenses does not exceed 1000 yuan (US $148.19) per year. The patient pays any additional costs. For outpatients with public health care, the treasury reimburses 80% of the expenses, and the patient pays 20%.

To alleviate the problem of long payment queues, many hospitals in China have established various mobile apps that outpatients without health insurance could use to pay bills [8]. However, medically insured outpatients still need to pay manually at the checkout window. There are many outpatients in the tertiary hospitals in China, >50% of whom are insured [9]. As medically insured outpatients cannot make payments on the web, a large number of medically insured outpatients have to spend a long time waiting in a line at the manual pay window, which can even take up to an hour during peak hours. The inability to pay on the web through medical insurance plan has long been seen as one of the biggest obstacles to the development of a mobile medical care system [10]. The *Action Plan for Further Improvement of Medical Services (2018-2020)* formulated by the National Health and Family Planning Commission of China [11] suggested that medical institutions should use internet technology to provide patients with convenient health care services such as mobile payment. Further challenges presented by Albreht et al [12] included improving the efficiency of health system management by reforming procurement and provider payment systems. Preventing and controlling COVID-19 require fewer on-site hospital personnel, so shortening long queues at the cashier window and quickly shunting patients are particularly important.

Our hospital has more than 10,000 outpatients every day. According to the hospital satisfaction survey conducted in our hospital by a third party in the fourth quarter of 2020, the overall satisfaction score in our outpatient clinic was 87.10 points, and the satisfaction score for waiting time was 84.97 points, which were the lowest scores across 9 dimensions. Therefore, shortening the waiting time is essential for improving outpatients’ satisfaction. Our hospital has implemented a comprehensive nonemergency appointment registration system [13] starting from October 2015. Since then, 93% of our patients have made appointments through mobile phones. Hence, in the era of the popularization of smartphones, it is of urgent importance to use the innovation of information technology to achieve mobile payment for medical insurance.

Guangzhou Women and Children’s Medical Center is the national children’s medical center in South Central China and the largest women and children’s hospital in South China. Its outpatient clinic achieved Healthcare Information and Management Systems Society Stage 7 status in 2016. Our hospital has implemented mobile medical insurance payment since January 2021, aiming to shorten patients’ waiting time and thus becoming one of the first pilot hospitals of Guangzhou Municipal Human Resources and Social Security Bureau and one of the first hospitals in China to effectuate medical insurance mobile payment.

### Objective

This study aimed to explore the effect of mobile payment of medical insurance on patients’ payment waiting time, waiting time for medical consultation, satisfaction with registration payment experience, the proportion of those dissatisfied with payment, outpatient satisfaction, and outpatient volume. We hypothesized that medical insurance mobile payment for outpatient services would be better than on-site manual payment requiring patients to queue at the cashier window.

## Methods

### Research Design and Subjects

This was a historically controlled study. We compared the effects of 2 payment methods before and after the implementation of mobile phone payments for medically insured outpatients. The traditional, medically insured outpatients included those making on-site manual payments. Since the implementation of medical insurance mobile payment in January 2021, medically insured outpatients in our hospital could pay their bills through their phones after visiting a doctor without needing to queue at the cashier window to make a manual payment. The operation guide protocol was as follows: enter the WeChat official account of “Guangzhou Women and Children’s Medical Center,” then followed by “outpatient service payment,” “pay immediately,” and “pay with a medical insurance card” (Multimedia Appendix 1 shows a screenshot of medical insurance mobile payment).

In the outpatient departments of Chinese hospitals, mobile phone payment, self-service machines, and manual payment are all used. Self-paying patients could choose any of the above modalities to make the payment, with mobile phone payment being the fastest solution. Outpatients in most hospitals can only make payments in person. To make the web-based payment via mobile phone for medically insured outpatients, the close cooperation of multiple departments of the hospital, including the outpatient department, information technology department, medical insurance department, Guangzhou Medical Insurance Bureau, and so on, is required. We promoted outpatient mobile payment of medical insurance through a variety of ways, including pushing the operation procedure of outpatient mobile payment of medical insurance to patients and conducting publicity on the official website, public hospital account, hospital display screen, nurse stations in each outpatient department, and in front of the fee collection office.

Currently, Chinese hospitals provide various modalities of making payments. To support older adults and other people with low information literacy to adapt to the development of modern information technology, our hospital established a special counter for older adults to make payments with staff to help and teach them.

Inclusion criteria were the following: (1) the participant had records in the hospital information system during the study period; (2) the participant used mobile payment for medical insurance; and (3) the participant had good communication skills and agreed to participate in this study.

Exclusion criteria were the following: (1) the participant had incomplete registration or enrollment data; and (2) the participant had poor communication skills.

### Patient Grouping

Medically insured outpatients who queued at the cashier window for manual payment from January 2020 to December 2020 were recruited as the control group. Medically insured outpatients who used smartphones to make a payment from January 2021 to December 2021 were recruited as the research group.

### Data Collection

The number of outpatients was extracted from our hospital’s management decision support system (business intelligence system). In addition, data such as satisfaction with waiting time, satisfaction with the registration payment experience, overall satisfaction, and the proportion of patients who believed that the waiting time should be shortened were extracted from the quarterly survey of total satisfaction of outpatients in our hospital. The data collection period was from 1 year before to 1 year after the smartphone-based medical insurance payment implementation. The satisfaction survey was conducted through computer-assisted telephone and short SMS text messages. The satisfaction survey is routinely collected quarterly. A stratified random sampling method was used for the survey. At least 1111 cases were collected quarterly. Based on sample size calculations, 1111 cases were queried, and the results showed that the respondents were representative.

The calculation of sample size for the degree of survey satisfaction was made according to Sun and Yong [14]. The equation for designing sample size in the current study was n = *Z*^2^ × *P*(1 – *p*) / *E*^2^, where *E* is the acceptable sampling error and *Z* is the confidence level of standard error; when the confidence coefficient was 95%, the correspondingly *Z* was 1.96. When the constituent ratio was *P*=0.806 and *E*=0.05, the minimum sample size was 999. With a 10% invalid response rate, the sample size was 1110.

The total patient satisfaction was the first-level indicator. It consists of 2 parts. The first part has a weight of 70%, and there are 9 second-level indicators including service efficiency, service quality, hospital environment, service attitude, medical ethics, medical technology service, pharmacy service, medical expense, and complaint channel. Furthermore, 20 third-level indicators were assessed. The 20 indicators included the waiting time of patients for payment, patient satisfaction with the waiting time, patient satisfaction with registration payment experience, the proportion of patients dissatisfied with payment, and so on. The responses to third-level indicators were categorized using the 5-point Likert scale, in which a higher score indicated a higher degree of agreement, that is, 5 points indicated a complete agreement and 1 point indicated a complete disagreement. This survey defined a score of 1-2 points as “complaining,” 3 points as “neutral,” and 4-5 points as “satisfied.” The second part is the general impression of the patient for the hospital, using a 10-point scale, with a weight of 30%. The survey results were calculated by mean, and the overall situation and index scores of hospitals and departments were analyzed by computer software.

The total satisfaction was calculated by summing the scores of 2 parts after multiplying them by their corresponding weights: score of the first part (70%) and the score of the second part (30%). The percentage score of the first part was equal to the mean values of the second-level indicators multiplied by 20. The score of the second-level indicators were the mean values of the third-level indicators [9,15].

### Statistical Analysis

SPSS software (version 23.0; IBM Corp) was used to perform statistical analyses. Independent sample 2-tailed *t* test was used to compare waiting time for payment, satisfaction with waiting time for medical consultation, and overall satisfaction. Paired sample 2-tailed *t* test was used to compare monthly outpatient visits. The chi-square test was used to compare the percentage of patients dissatisfied with payment. *P*<.05 represented statistical significance.

### Ethics Approval

This study was reviewed and approved by the Research Ethics Committee of Guangzhou Women and Children’s Medical Center (approval number SFE-2022-193A01), and the patients or their guardians signed the informed consent form. This was an observational study based on anonymous surveys. The study data were anonymous.

## Results

### Respondent Characteristics

A total of 9260 questionnaires were released, and 9015 were collected, indicating an effective recovery rate of 97.35%. Of the 9015 respondents, female respondents accounted for 79.48% (n=7165), and male respondents accounted for 20.52% (n=1850). Guangzhou urban area respondents accounted for 66.97% (n=6037), Guangzhou suburb area respondents accounted for 12.51% (n=1128), and respondents from outside Guangzhou accounted for 20.52% (n=1850). Respondents aged ≤20 years accounted for 5.85% (n=527), respondents aged 21-40 years accounted for 75.88% (n=6841), respondents aged 41-60 years old accounted for 17.82% (n=1606), and respondents aged >60 years old accounted for 0.45% (n=41).

### The Waiting Time of Patients for Payment

The mean waiting time before the implementation of the mobile payment for health insurance was 45.28 (SD 10.35) minutes. After implementation, this was significantly reduced (mean 1.02, SD 0.25 min; t_9014_=53.396; *P*<.001). The mean time saved was 44.26 (SD 0.829) minutes.

### Patient Satisfaction With the Waiting Time

As shown in Table 1, after implementing medical insurance mobile payment, the patients’ satisfaction with the waiting time for medical consultation was significantly improved (*P*<.001).

**Table 1:** Comparison of patients’ satisfaction with the waiting time before and after mobile payment for medical insurance.

	Before implementation, mean (SD)	After implementation, mean (SD)	*t* test (*df*)	*P* value
First quarter	82.60 (2.59)	90.67 (3.63)	–60.65 (2244)	<.001
Second quarter	81.98 (3.21)	90.61 (3.81)	–58.25 (2260)	<.001
Third quarter	81.89 (3.18)	91.10 (3.35)	–67.05 (2261)	<.001
Fourth quarter	81.85 (3.37)	89.04 (3.12)	–52.47 (2246)	<.001
Overall satisfaction	82.08 (3.17)	90.36 (3.45)	–118.65 (9014)	<.001

### Patient Satisfaction With Registration Payment Experience

As shown in Table 2, compared to before the implementation of mobile payment for medical insurance, the patient satisfaction score on registration payment experience was significantly improved after implementing the mobile payment system (*P*<.001).

**Table 2:** Comparison of the patient satisfaction score on registration payment experience before and after the implementation of mobile payment for medical insurance.

	Before implementation, mean (SD)	After implementation, mean (SD)	*t* test (*df*)	*P* value
First quarter	85.80 (3.57)	90.28 (3.63)	–29.48 (2244)	<.001
Second quarter	85.12 (3.31)	90.01 (3.27)	–35.33 (2260)	<.001
Third quarter	86.95 (2. 95)	92.00 (3.08)	–39.82 (2261)	<.001
Fourth quarter	84.39 (3.05)	89.40 (3.02)	–39.12 (2246)	<.001
Overall satisfaction	85.57 (3.44)	90.42 (3.27)	–68.61 (9014)	<.001

### The Proportion of Patients Dissatisfied With Payment

As shown in Table 3, compared to before the implementation of mobile payment for medical insurance, the proportion of patients who were dissatisfied with payment was significantly lowered after implementing mobile payment (*P*<.001).

**Table 3:** Comparison of the proportion of patients who were dissatisfied with payment before and after the implementation of mobile payment for medical insurance.

		Before implementation	After implementation	*P* value
**Proportion of patients who were dissatisfied, n/N (%)**				
	First quarter	88/1124 (7.83)	18/1121 (1.61)	<.001
	Second quarter	109/1133 (9.62)	11/1128 (0.98)	<.001
	Third quarter	119/1128 (10.55)	13/1134 (1.15)	<.001
	Fourth quarter	147/1125 (13.06)	11/1122 (0.98)	<.001
Overall proportion of patients who were dissatisfied (%), mean (SD)		10.27 (2.18)	1.19 (0.30)	<.001

### The Overall Satisfaction of Outpatients

As shown in Table 4, compared to before the implementation of mobile payment for medical insurance, the total satisfaction of outpatients was significantly improved after they could use mobile payment.

**Table 4:** Comparison of the overall satisfaction of outpatients before and after the implementation of the mobile payment for medical insurance.

	Before implementation, mean (SD)	After implementation, mean (SD)	*t* test (*df*)	*P* value
First quarter	84.54 (3.38)	90.26 (3.17)	–41.35 (2244)	<.001
Second quarter	87.74 (3.04)	90.06 (3.04)	–18.14 (2260)	.005
Third quarter	88.27 (2.81)	90.52 (3.73)	–16.20 (2261)	.01
Fourth quarter	87.10 (3.13)	89.06 (3.35)	–14.33 (2246)	.03
Overall satisfaction	86.91 (3.23)	89.98 (3.31)	–44.57 (9014)	<.001

### The Number of Outpatient Visits

One year before the implementation of the new payment system, the monthly outpatient volume was mean 248,105.58 (SD 89,280.76) outpatient visits. Compared with the previous year, the number of outpatient visits per month was increased after launching the new payment system (mean 303,194.75, SD 53,773.12; t_11_=2.414; *P*=.03). The difference was mean 55,089.17 (SD 79,050.47) outpatient visits.

### On-site Manual Cashiers

Compared to before the implementation of mobile payment for medical insurance outpatients, implementing mobile payment improved payment efficiency, and the number of the on-site cashier has decreased by 9.

## Discussion

### Principal Findings

The present study analyzed the impact of smartphone-based mobile payment system for outpatients with medical insurance. The results showed that after the implementation of mobile payment for outpatients with medical insurance, the waiting time of patients to make payment was significantly shortened; the patients’ satisfaction with the waiting time and registration payment experience significantly improved; the proportion of patients dissatisfied with payment significantly reduced; the overall satisfaction of outpatients improved; and the number of monthly outpatient visits significantly increased. Furthermore, our hospital was invited to share the experience of mobile payment for medical insurance with colleagues from other hospitals across China.

Our findings revealed that implementing mobile payment could *significantly* shorten the waiting time for payment for outpatients with medical insurance, significantly improve patients’ satisfaction, and significantly increase the number of outpatient visits. This substantially shortened the long queues of medically insured outpatients waiting to make manual payments at the counter, especially during the COVID-19 epidemic, which is conducive to the rapid triage of outpatients in hospitals. This study addressed the issues discussed in previous research. For example, in the study by Li et al [16] in China, the respondents suggested strengthening the informatization support for the reform of medical insurance payment modes to provide more self-service supports for those insured. Liu et al [17] proposed that hospital payment reform, reimbursement policy reform, and hospital innovation should be further evaluated. The obstacles to medical payment include the predominant fee-for-service payment model, which does not reimburse for web-based visits [18]. A survey conducted by Suárez-Obando et al [19] in Colombia showed that 93% of patients possessed a mobile phone. It was necessary to conduct more research on medical health apps to ensure the quality and reliability of mobile health apps [20]. Mobile health is currently experiencing explosive growth and could be a groundbreaking innovation that would change the face of health care in the future [21].

The results of this study were not consistent with some previous findings. The patients’ waiting time in our study was shorter than that of insured patients in a tertiary hospital in Nigeria, as reported by Opurum et al [3]. They proposed that there were significant differences in mean total idle time during outpatient visits (65.7 min) and total time spent (106.6 min), and the results favored insured patients. Other scholars explored shortening the patient waiting time to improve their satisfaction in relation to different aspects. Ala et al [22] accomplished a comprehensive review of appointment scheduling and proposed several methods to shorten the patients’ waiting time in health care centers. Information technology has been used to optimize the identification process for outpatients having blood drawn and improve patients’ satisfaction [23]. Murteira et al [24] investigated other effects in terms of dispensing of hospital-specific medicines to community pharmacies, waiting time reported by participants, and pharmacist availability.

The implementation of mobile payment for medical insurance has improved payment efficiency and substantially decreased the number of patients queuing in front of the cashier window, which led to the number of on-site cashiers being decreased by 9. The informatization operation of our hospital could save labor costs, which is consistent with the view of improving efficiency proposed by other researchers, who also suggested providing a higher quality of care at lower cost [25,26]. Albreht et al [12] proposed to enhance health system management efficiency through the reform of purchasing and provider payment systems.

The present study showed that monthly outpatient visits increased compared with the previous year, which may be related to the fact that the medically insured outpatients can use their mobile phones to make payments, as this shortens their waiting time and improves their satisfaction. Consequently, patients were more willing to visit our hospital.

### Strengths and Limitations

The strengths of insured outpatients using mobile payment explored in this study included avoiding waiting in long queues at the cashier window, significantly shortening the waiting time in the hospital, and improving patient satisfaction. As our hospital is specialized in women and children’s care, the patients who visit our hospital normally come in the company of family members or guardians. This study focused on how to save time for not only patients but also those accompanying the patients. In addition, with the help of information technology, the smartphone-based mobile payment for medical insurance optimized the procedures for medical treatment, replaced the on-site manual payment process, and improved work efficiency, leading to the reduction of cashier personnel and labor costs.

The present study also has some limitations. This study was not a randomized controlled trial but a historically controlled study. There may be individual differences due to different cases. There may be bias and confounding factors due to the large differences between the 2 stages. We analyzed patients for 2 consecutive years within the same time span for the study, and the patients were similar in gender, age, and other characteristics. Only one hospital was surveyed; thus, the collected data and indicators were limited. The involvement of more medical centers and a further increase in the number of indicators collected are needed in the future. Additionally, this study was conducted in a low-income country with a medical insurance system. Mobile payment for medical insurance requires advanced information technology, demanding the integration of a hospital information system, payment system, and medical insurance information system.

### Conclusion

The implementation of the mobile payment significantly shortened the waiting time of outpatients with medical insurance to make the payment; improved patient satisfaction with the waiting time and registration payment experience and overall satisfaction; reduced the proportion of patients who were dissatisfied with payment and the number of cashiers in the hospital; and significantly increased monthly outpatient volume. Therefore, this mobile payment method is worth promoting, especially in low-income countries. Future research will continue to focus on extending web-based mobile inpatient payment and leveraging information technology to improve payment efficiency and optimize the payment process, making it more convenient for patients.

## Appendix

Multimedia Appendix 1 Screenshot of medical insurance mobile payment.

## Data Availability

The data sets generated during and/or analyzed during the current study are available from the corresponding author upon reasonable request.
