# Supplementary figures and images for: The Effect of Mobile Payment on Payment Waiting Time for Outpatients With Medical Insurance: Historically Controlled Study
Source: JMIR Form Res. 2023 Jan 26;7:e43167. doi: 10.2196/43167 (PMC9912152; doi:10.2196/43167)

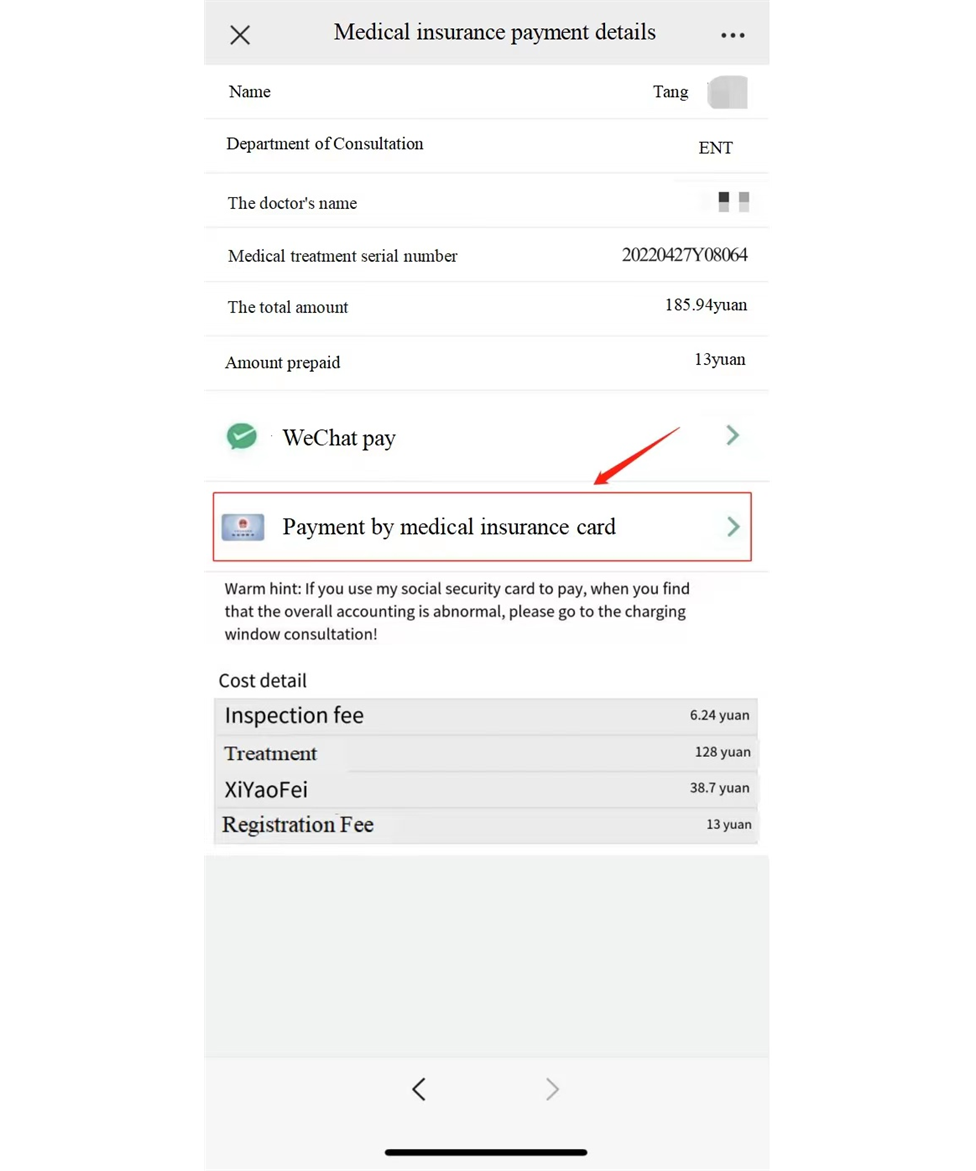

Supplement: Multimedia Appendix 1 [file formative_v7i1e43167_app1.png]
